# Associations Between Dog Breed and Clinical Features of Mammary Epithelial Neoplasia in Bitches: an Epidemiological Study of Submissions to a Single Diagnostic Pathology Centre Between 2008–2021

**DOI:** 10.1007/s10911-023-09531-3

**Published:** 2023-03-24

**Authors:** Grace Edmunds, Sam Beck, Kedar Umakant Kale, Irena Spasic, Dan O’Neill, David Brodbelt, Matthew J. Smalley

**Affiliations:** 1 Bristol Veterinary School, Langford House, Langford, Bristol, BS40 5DU UK; 2 VPG Histopathology (Formerly Bridge), Horner Court, Horfield, Bristol, BS7 0BJ UK; 3 Present Address: Independent Anatomic Pathology Ltd, Bath, UK; 4 School of Computer Science and Informatics, Cardiff University, Cardiff, CF24 4AG UK; 5 The Royal Veterinary College, North Mymms, Hatfield, Herts AL9 7TA UK; 6 School of Biosciences, European Cancer Stem Cell Research Institute, Cardiff University, Hadyn Ellis Building, Maindy Road, Cardiff, Wales CF24 4HQ UK

**Keywords:** Canine, Mammary, Adenoma, Carcinoma, Complex, Mixed, Cancer

## Abstract

Mammary cancer is one of the most common neoplasms of dogs, primarily bitches. While studies have been carried out identifying differing risk of mammary neoplasia in different dog breeds, few studies have reported associations between dog breeds and clinical features such as number of neoplastic lesions found in an individual case or the likelihood of lesions being benign or malignant. Such epidemiological studies are essential as a foundation for exploring potential genetic drivers of mammary tumour behaviour. Here, we have examined associations between *breed*, *age* and *neuter status* and the odds of a diagnosis of a mammary epithelial-origin neoplastic lesion (as opposed to any other histopathological diagnosis from a biopsied lesion) as well as the odds of a bitch presenting with either a single mammary lesion or multiple lesions, and the odds that those lesions are benign or malignant. The study population consisted of 129,258 samples from bitches, including 13,401 mammary epithelial neoplasms, submitted for histological assessment to a single histopathology laboratory between 2008 and 2021.

In multivariable analysis, *breed*, *age* and *neuter status* were all significantly associated with the odds of a diagnosis of a mammary epithelial-origin neoplastic lesion. Smaller breeds were more likely to receive such a diagnosis. In cases diagnosed with a mammary epithelial neoplasm, these three factors were also significantly associated with the odds of diagnosis with a malignant lesion and of diagnosis with multiple lesions. Notably, while neutered animals were less likely to have a mammary epithelial neoplasm diagnosed, and were less likely to have multiple neoplasms, they were more likely to have malignant disease. Exploration of the patterns of risk of developing malignant disease, or multiple lesions, across individual breeds showed no breed with increased odds of both outcomes. Breeds with altered odds compared to the Crossbreed baseline were either at increased risk of malignant disease and decreased risk of multiple lesions, or vice versa, or they were at significantly altered odds of one outcome with no change in the other outcome. Our analysis supports the hypothesis that age, neuter status and intrinsic biological and genetic factors all combine to influence the biological heterogeneity of canine mammary neoplasia.

**Supplementary Information:**

The online version contains supplementary material available at 10.1007/s10911-023-09531-3.

## Introduction

The mammary gland is a common site for neoplasia in dogs [1]. For example, an insurance register study of female dogs in Sweden (where only a small proportion of bitches, approximately 7%, are neutered) [2] reported an overall incidence of 111 cases (including both benign and malignant neoplasms) per 10,000 dog years at risk (DYAR). Incidence increased with age and varied by breed [3]. A study of cancer rates in dogs insured with a UK pet insurance company (in the UK approximately 60% of female dogs are neutered) [4] reported an estimated age-standardised incidence of 205 cases (benign and malignant) / 100,000 dogs / year [5]. The latter study did not distinguish between male and female dogs.

As with human breast cancer, mammary cancer is more common in female than in male dogs [6, 7] and exposure to female hormones is likely important for the aetiology of mammary neoplasia in bitches, with neutering suggested to reduce risk of occurrence [8–11] (D. Varney, D. O'Neill, M. O'Neill, D. Church, A. Stell, S. Beck, M. Smalley and D. Brodbelt: The epidemiology of mammary tumours in bitches under veterinary care in the UK in 2016, submitted). A recent systematic review questioned the strength of the epidemiological evidence supporting an association between neutering and mammary tumour risk in dogs, in particular relating to the timing of neutering [12], however, the data are overall consistent that neutering (particularly at a younger age) reduces mammary tumour risk [13]. If an entire animal develops a mammary tumour, neutering combined with mastectomy significantly increases survival compared to neutering alone [14] although estrogen may have a protective effect in animals with estrogen receptor (ER) negative tumours [15], so neutering may be counterproductive in these cases. Like human breast cancer, canine mammary tumours which lack hormone receptors tend to be more aggressive [16–18]. Other features of similarity between canine and human mammary tumours include their age of onset (usually middle-aged to elderly individuals), shared environmental exposures, the opportunity and common motivation to treat with curative intent and their potential for metastasis via the lymphatic system. Furthermore, the presence of histological subtypes with prognostic significance [19–22] make canine mammary tumours an ideal model system within which to investigate mammary tumour heterogeneity.

The key strength of the dog as a disease model, however, lies primarily in the genetics of the current breed structure of dogs. Modern dog breeds have been highly selectively inbred to accentuate particular and desired phenotypic and/or behavioural features. In the process, many individual breeds have become predisposed to particular diseases or pathological conditions, either as a direct result of the phenotypic feature being selected for (e.g. brachycephaly and skin fold dermatitis) [23] or because a risk allele(s) for the pathology is/are in linkage disequilibrium with the genetic variants responsible for the selected features (e.g. Dachshunds and Lafora Disease). Differential breed-associated disease predisposition also affects cancer risk in dogs [2, 5, 7, 9, 24–29]. For example, we recently demonstrated that large dogs are at significantly higher risk of osteosarcoma, while brachycephalic dogs (with flat faces, such as bulldogs) are at significantly lower risk [30]. Breed associations with mammary cancer risk have also been demonstrated [3, 7, 9, 24–27]. We have also recently identified an increased risk of mammary neoplasia in English Springer Spaniels, Lhasa Apso and Staffordshire Bull Terriers (D. Varney, D. O'Neill, M. O'Neill, D. Church, A. Stell, S. Beck, M. Smalley and D. Brodbelt: The epidemiology of mammary tumours in bitches under veterinary care in the UK in 2016, submitted). The increased risk of mammary cancer in English Springer Spaniels has previously been noted [3, 25], with a suggested link to *BRCA1* and *BRCA2* polymorphisms [31].

However, there is little information on whether the breed of dog in which a mammary neoplasm develops influences the biology of that tumour, over and above any effect of the risk of that tumour forming in the first place. If such breed-biology associations were elucidated, they would validate canine mammary tumours as a model system for investigating the genetic drivers of mammary tumour heterogeneity.

Dogs with mammary neoplasia can present with single palpable masses or multiple masses (although anecdotally ‘single masses’ can be found to be in fact composed of more than one lesion upon histological examination). The number of lesions found, and their classification as benign or malignant, can determine how aggressively the animal is clinically managed. Surgical interventions may range from simple lumpectomy of a benign adenoma to complete removal of the mammary chain, sometimes bilaterally and in conjunction with ovario-hysterectomy [1]. Here, we have investigated associations between epidemiological risk factors (*breed*, *age* and *neuter status*) with two pathological features of canine mammary tumours submitted for histology: (i) the number (single or multiple) of lesions on the submitted mammary chain and (ii) the histotypes (benign or malignant) reflected by those single or multiple lesions. Data were generated from a large archival record of 129,258 biopsy samples with associated clinical data from female domestic dogs. Samples were submitted to a single diagnostic centre between 2008 and 2021, and 13,401 had a confirmed diagnosis of at least one epithelial-origin mammary neoplasm.

We find a significant association in the current dataset, between bitch *breed*, *age* and *neuter status* and the odds of being diagnosed with a mammary epithelial neoplasm as opposed to a diagnosis for any other condition. Furthermore, *breed*, *age* and *neuter status* were also significantly associated with whether a case presented with only a single mammary epithelial neoplastic lesion or with multiple lesions, as well as with whether benign or malignant disease was diagnosed. Of particular note, neutered animals had a small but statistically significant increase in odds of diagnosis with a malignant mammary epithelial neoplasm. Our findings will form the basis to develop testable hypotheses addressing the genetic drivers of mammary tumour biological heterogeneity.

## Methods

### Study Samples and Data Processing

The study was approved by Cardiff School of Biosciences Research Ethics Committee (20–05-01). Information on samples submitted to VPG Histopathology, Bristol, UK (https://vet.synlab.co.uk/histopathology/) between 2008 and 2021, with consent for research use, were retrieved by a search of the VPG Histopathology block archive database for all records listed as canine in origin. In total, 300,592 records were downloaded as a Microsoft Excel spreadsheet with the following information: case number, breed, age at sample submission (years and months), sex and neuter status, histological diagnosis. The latter was a semi-standardised free-text field which contained in some cases a single diagnosis, with information on severity and location, whilst in other cases there were multiple diagnoses of samples from the same case, which might have come from the same or different anatomical locations. Therefore, each sample record was a record of an individual submission from a single animal, but might have included biopsies from multiple locations. We cannot exclude that, given the extended period of time over which samples were submitted, an animal might have had samples submitted for analysis more than once. Furthermore, as different pathologists had reported on cases over the 11-year period, terminology was not uniform even for identical lesions.

Data were cleaned in Microsoft Excel (2013, Microsoft Corp.), and Rstudio™ using the packages listed in the R script (available at https://github.com/ge8793/Mammary_Public_Data/blob/main/Mammary_Public_Code_Nov22.R). Records with duplicate case numbers were identified and, where the duplicate was clearly the same record entered twice, the entry with the least information (typically lacking diagnostic information) was deleted. Where duplicates were clearly different submissions which had been given the same number in error, both were kept but one was given a letter as a prefix to the sample number to make it uniquely identifiable. Any submission for which either the breed or diagnostic information was absent or ambiguous was excluded. Any submissions from wild canids were excluded. Any submissions with the histological information not in English were excluded. Finally, any submission from a male dog or a submission for which the sex was unknown was excluded. That left 129,258 submissions from female dogs where breed information and histology/location of their lesion(s) was known. See Table S1 for the details of all submissions.

Breed descriptions were cleaned to remove typographical errors and standardise terminology as detailed previously [30]. Some similar breeds were grouped together under ‘unspecified/other’ both to simplify analysis and allow for the fact that full breed designations had not always been recorded (e.g. ‘Griffon Bruxellois’ and ‘Basset Griffon Vendeen’ were grouped as ‘Griffon (unspecified/other)’; ‘Springer Spaniel’ and ‘English Springer Spaniel’ were grouped as ‘Springer Spaniel (unspecified/other)’). Finally, breeds with < 130 individuals (i.e. 0.001%) of the total, were grouped together as ‘Other Purebred’. All ‘designer’ crosses (purposely bred crossbreeds with contrived names generated from two or more purebred breed terms) were included in the Crossbreed group. The cleaned and standardised breed data comprised the *breed* variable.

Neuter status was recorded as female entire, female neutered or female, neuter status unknown. *Age* was taken as the date of sample submission and then was categorised as: < 3.0, 3.0 to < 6.0, 6.0 to < 9.0, 9.0 to < 12.0 and ≥ 12.0 years. Bitches for which there was no age data recorded were maintained in the analysis, meaning that confidence intervals and *p*-values were also generated for the ‘age unrecorded’ category..

Following the initial clean-up, the diagnosis information was searched for the term ‘mammary’. This identified 13,708 cases in which mammary tissue had been submitted for diagnosis (in some cases alongside samples from other tissues; Table S2).

Next, we developed a Natural Language Processing script in Python using the Jupyter Notebooks environment on the Kaggle platform (https://www.kaggle.com/) to extract diagnostic terms from the records of these 13,708 samples in an automated, standardised manner and export these to a CSV file. This script was based on the conditional random fields approach [32, 33] and also MetaMap (https://lhncbc.nlm.nih.gov/ii/tools/MetaMap.html) to extract key characteristics and map these to the National Library of Medicine's Unified Medical Language System [34] Metathesaurus. The script is available as a Supplementary Data File.

The extracted histological diagnoses underwent additional clean-up using Excel tools to standardise histological terminology according to the Surgical Pathology of Tumours of Domestic Animals [35] system, first into ductal and non-ductal tumours and then non-ductal tumours were further classified as simple, non-simple and special types (Table S3) as well as intermediate forms (e.g. carcinoma, tubulopapillary to solid). Extracted data were also reviewed to ensure that where a case had been diagnosed with multiple mammary lesions of epithelial origin, all had been captured. At this stage, a further 307 cases were excluded (Table S1: ‘Triaged out’) for one of several reasons: no neoplastic lesion had been identified (e.g. only mammary hyperplasia); only carcinoma *in situ* was reported (the status of carcinoma *in situ* in the dog is unclear but current guidelines suggest it should not be classified as a tumour) [35]; a mammary lymph node with metastatic carcinoma was reported but the primary site was unknown; the only neoplasm in the mammary tissue was not of epithelial origin (e.g. fibrosarcoma; note, where a sarcoma was recorded as arising in a benign mixed tumour, the case was included as a benign epithelial lesion and not triaged). This left 13,401 cases with a standardised diagnosis of one or more mammary epithelial neoplasms and 115,550 samples with a diagnosis (of any condition) involving a location other than the mammary gland. Table S4 gives the categorisation of all cases as mammary / not mammary / triaged out.

Mammary epithelial cases were then categorised as having malignant lesions (which could include only malignant lesions or both benign and malignant lesions) or benign lesions and as having only a single diagnosed lesion or multiple lesions (which could include multiple mammary epithelial neoplastic lesions of the same type or different types of mammary epithelial neoplastic lesion). Table S5 gives the categorisation of the mammary cases as benign/malignant and single/multiple as well as the breakdown of the histology of each case.

Finally, data validation back to the original downloaded dataset was carried out to ensure that the diagnostic information and categorisation was linked to the correct submission number and signalment data.

### Statistical Analysis

It was not possible to carry out an accurate power calculation *a priori* for this study, because there were no previous studies with which to estimate the effect sizes associated with breed and the likelihood of single versus multiple mammary epithelial tumours in dogs. Therefore, we chose to retrospectively determine the power of the study for one breed, Cocker Spaniel (Unspecified/Other) (1001 cases diagnosed with mammary epithelial neoplastic lesions), which we found to have a small but still significant increased odds of developing multiple lesions compared to Crossbreeds in multivariable analysis (OR 1.37, 95%CI 1.24 – 1.84) and one breed, Setter (Unspecified/Other) with very few cases (21) but which had the highest odds in this analysis (OR 2.55, 95%CI 1.06 – 6.11). Using the tool available at https://www.openepi.com/Power/PowerCC.htm [36] with methodology from Edmunds and colleagues [30, 37], we calculated that for Cocker Spaniel (Unspecified/Other) we had 98% power while for Setter (Unspecified/Other) we had 50—60% power. Obviously, where breeds have fewer representatives in the dataset, or effect sizes are smaller, the likelihood of a type-I error is increased.

Statistical methodology was derived from previous studies which quantified associations between demographic risk factors and cancer in dogs [30, 38] (Varney D, O'Neill D, O'Neill M, Church D, Stell A, Beck S, Smalley M, Brodbelt D: The epidemiology of mammary tumours in bitches under veterinary care in the UK in 2016, submitted). Associations between risk factors (*breed*, *age*, *neuter status*) and histological diagnosis of a mammary lesion, and, of the mammary tumours only, associations with the development of multiple mammary epithelial neoplastic lesions or a diagnosis of malignant mammary epithelial neoplastic disease were determined using binary logistic regression modelling (glm-logit function, R-stats package) [30, 39]. A global *p*-value for each was calculated using ANOVA to compare the univariable regression model with a null model derived from the proportions of mammary neoplastic diagnoses, multiple mammary epithelial neoplastic lesions and malignant mammary epithelial neoplastic disease in a comparator group (Crossbreeds for the *breed* variable; age < 3 years for the *age* variable; entire animals for the *neuter status* variable). The lmtest package was used to generate global *p*-values in multivariable modelling [30, 40]. Risk factors that were significantly associated with the outcomes in univariable modelling (*p* < 0.20) were taken forward in multivariable modelling that used a manual backwards stepwise elimination approach [30, 41]. Final model statistical significance was set at the 5% level. The quality of the final multivariable model for each outcome was determined using the area under the ROC curve [30, 41]. Pairwise interactions were not evaluated for all variables in the final models but instead evaluation for interaction was restricted to variables deemed to have a relevant biological interaction. Descriptive statistics from the analyses are given in Table S6 and all results are provided in Table S7.

The association between odds of a mammary neoplasia being reported in a breed and the mean body weight for a female of that breed (taken from VetCompass estimates of average breed mass) [30, 42] (https://www.rvc.ac.uk/VetCOMPASS) was explored with simple linear regression in GraphPad Prism (GraphPad Software LLC). Analysis of distribution of non-simple versus simple tumours was carried out using Fisher’s exact test in GraphPad Prism on the distribution of the independent categorical variables between single and multiple mammary epithelial neoplastic lesion cases.

## Results

### Features of the Study Analysis Sample

**Fig. 1:**
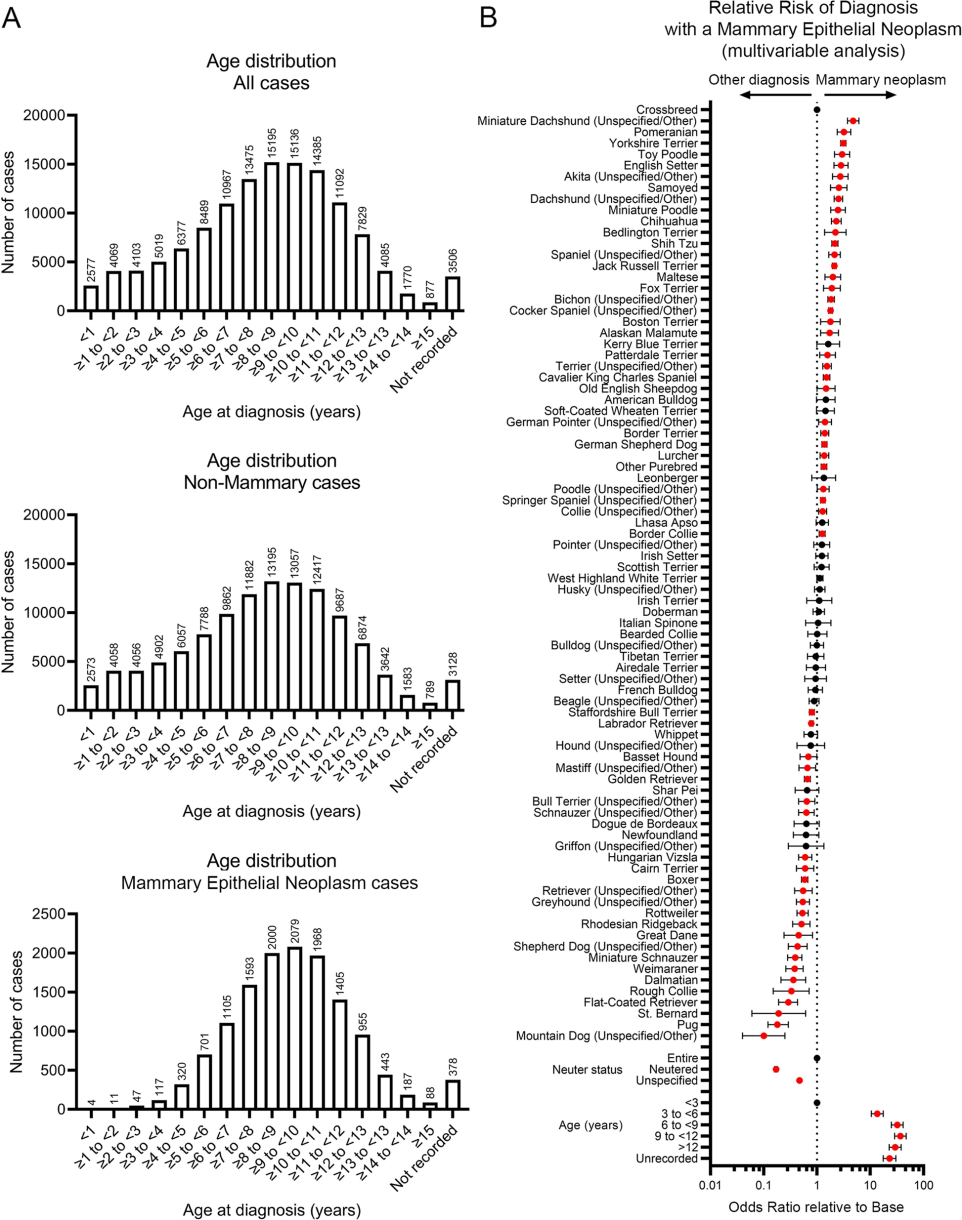
Presentation of canine mammary neoplasia. **A** Age distribution of all cases in study population (top), non-mammary cases (middle) and cases diagnosed with mammary epithelial neoplasia (bottom). **B** Forest plot showing results of multivariable analysis of *breed*, *neuter status* and *age* relationship of study population to odds of a diagnosis of a mammary epithelial neoplasm versus any other histological diagnosis of disease. Odds ratios and 95% confidence intervals are shown. Variables showing a significant difference from base reference value (Crossbreed for *breed*, Entire for *neuter status*, < 3 years for *age*) are indicated in red

The study population consisted of 129,258 female dogs with a sample submitted for routine histological diagnosis of any lesion between 2008 and 2021. Of these, 13,401 cases had received a diagnosis of one or more mammary epithelial neoplasms (in some animals, with other additional pathology not discussed here) and 115,550 cases with a diagnosis (of any condition including both neoplastic and non-neoplastic disease) involving a location other than the mammary gland. Among the study population, the most common breeds overall were Crossbreed (22,660 submissions; 17.5%), Labrador Retriever (16,538 submissions; 12.8%), Staffordshire Bull Terrier (6,443 submissions; 5.0%), Cocker Spaniel (6,401 submissions; 4.9%), Jack Russell Terrier (5,879 submissions; 4.5%) and Springer Spaniel (5,581 submissions; 4.3%) and the median age of sample submission was 8 years and 2 months (interquartile range 6 years – 10 years 3 months). 5.2% of animals were entire, 61.4% were neutered, while neuter status was not recorded for 33.4% of animals (Tables S1, S4, S6 and Fig. 1A).

The most common breeds diagnosed with a mammary epithelial neoplasm were Crossbreed (1,979 cases; 14.8%), Labrador Retriever (1,288 cases; 9.6%), Jack Russell Terrier (1,123 cases; 8.4%), Cocker Spaniel (1,001 cases; 7.5%), Springer Spaniel (684 cases; 5.1%) and Yorkshire Terrier (531 cases; 4.0%). The median age of sample submission was 9 years (interquartile range 7 years 4 months – 10 years 9 months). 12.5% of animals were entire, 38.5% were neutered, while neuter status was not recorded for 49.0% of animals (Tables S2, S5, S6 and Fig. 1A).

The most common breeds diagnosed with a non-mammary condition were Crossbreed (20,681 submissions; 17.9%), Labrador Retriever (15,250 submissions; 13.2%), Staffordshire Bull Terrier (5,961 submissions; 5.2%), Cocker Spaniel (5,400 submissions; 4.7%), Springer Spaniel (4,897 submissions; 4.2%) and Jack Russell Terrier (4,756 submissions; 4.1%). The median age of sample submission was 8 years (interquartile range 5 years 8 months – 10 years 3 months). 4.4% of animals were entire, 64.1% were neutered, while neuter status was not recorded for 31.6% of animals (Tables S1, S4, S6 and Fig. 1A).

### Risk Factors Associated with Diagnosis of a Mammary Epithelial Neoplasm

In univariable analysis, there was a significant association of *breed* (*p* < 0.0001), *age* (*p* < 0.0001) and *neuter status* (*p* < 0.0001) with a diagnosis of a mammary epithelial neoplasm versus any other disease, following submission of a sample for analysis (Table S7). All factors were therefore taken forward into a multivariable model and again all factors were highly significant (all *p* < 0.0001) (Table S7). In the multivariable analysis, older age was significantly associated with higher odds of a mammary epithelial neoplasm diagnosis (3 to < 6 years old: Odds Ratio, OR 13.55, 95%CI 10.47 – 17.54; 6 to < 9 years: OR 32.09, 95%CI 24.92 – 41.32; 9 to < 12 years: OR 36.70, 95%CI 28.51 – 47.26; > 12 years: OR 29.33, 95%CI, 22.69 – 37.91). Animals which were neutered or for which neuter status was unspecified had significantly lower odds of a mammary epithelial neoplasm diagnosis compared to entire bitches (OR neutered 0.17, 95%CI 0.16 – 0.18; OR neuter status unspecified 0.47, 95% CI 0.44 – 0.5) (Table S7 and Fig. 1B).

Breeds including Miniature Dachshund (OR 4.77, 95%CI 3.74 – 6.09), Pomeranian (OR 3.2, 95%CI 2.39 – 4.29), Yorkshire Terrier (OR 3.12, 95%CI 2.78 – 3.49), Toy Poodle (OR 2.95, 95%CI 2.13 – 4.09) and English Setter (OR 2.82, 95%CI 2.08 – 3.83) had significantly higher odds of having a diagnosis of a mammary epithelial neoplasm than another disease, while Rough Collie (OR 0.33, 95%CI 0.15—0.71, Flat-Coated Retriever (OR 0.29, 95%CI 0.19—0.43), St. Bernard (OR 0.19, 95%CI 0.06—0.61), Pug (OR 0.18, 95%CI 0.12—0.29) and Mountain Dog (OR 0.1, 95%CI 0.04—0.25) had significantly lower odds of having such a diagnosis compared to Crossbreeds (Table S7 and Fig. 1B).

**Fig. 2:**
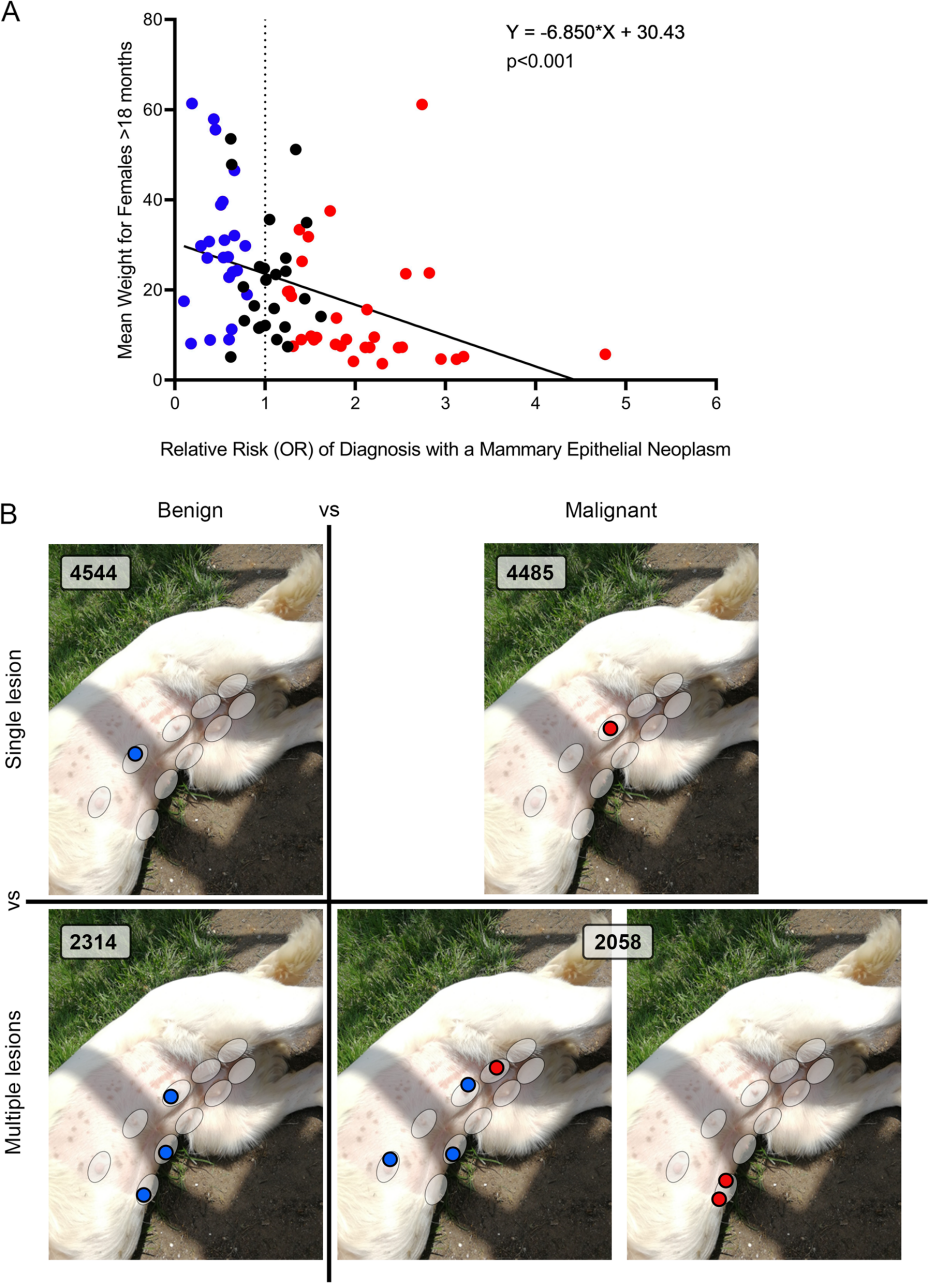
**A** Simple linear regression of mean breed body weight for purebred female dogs > 18 months old against odds of a diagnosis of mammary epithelial neoplasm relative to Crossbreed dogs. Each point represents one breed; points in blue/red represent breeds with significantly lower (blue) or higher (red) odds. There is a highly significant (*p* = 0.0004) inverse correlation between weight and diagnosis of a mammary epithelial neoplasm. See Table S8 for the details for each breed. **B** Cartoon indicating categories (single benign, multiple benign, single malignant, multiple malignant) into which dogs presenting with mammary tumours can be divided. Blue circles represent benign tumours, red cells malignant tumours. White ellipses indicate the five pairs of mammary fat pads

We noted that many of the higher risk breeds were smaller dogs while many of the lower risk breeds were larger dogs, although there were obvious exceptions (such as the English Setter and Pug). Individual level body weight data was not available in the current study. We therefore plotted the breed average body weight for females of each breed (see Methods) (Table S8) against the odds ratio of a mammary epithelial neoplasm diagnosis for that breed. There was a significant inverse correlation (*p* < 0.001) between the body weight and the odds of a diagnosis of mammary epithelial neoplasia (Fig. 2A).

### Features of the Cases Diagnosed with a Mammary Epithelial Neoplasm

Of the 13,401 female dogs in our sample diagnosed with one or more mammary epithelial neoplasms, 4544 cases (33.9%) were diagnosed with a single benign neoplastic epithelial mammary lesion. 2,314 cases (17.3%) were diagnosed with multiple benign lesions. 4,485 cases (33.5%) were diagnosed with a single malignant lesion while 2,058 cases (15.3%) were diagnosed with multiple lesions including one or more malignant neoplasms (Table S7 and Fig. 2B).

The most commonly diagnosed pathologies were benign mixed tumour (4,237 diagnoses), adenoma, complex (3,303 diagnoses), carcinoma, complex (1,714 diagnoses), carcinoma, simple (1,531 diagnoses), carcinoma, ductal (1,139 diagnoses) and adenoma, simple (928 diagnoses) (Table 1 shows the complete list of diagnoses). Note that in cases presenting with multiple lesions, each case could have more than one of these diagnoses and the exact number of lesions of each individual type was not necessarily counted (e.g. ‘multiple simple adenomas and simple carcinomas’). As a result, the overall proportions of different diagnoses cannot be calculated across the whole sample set. However, for cases in which only a single lesion was reported, the proportions of the different tumour types can be determined. In such single lesion cases, 25.55% (2,226) of tumours were benign mixed tumours, 14.84% (1,340) were complex adenomas, 11.39% (1,028) were simple carcinomas, 10.74% (740) were complex carcinomas, 8.00% (722) were ductal carcinomas, with all other histotypes being less than 5% of the total. The full list is given in Table 2.

**Table 1:** Numbers of histological diagnoses across all cases

**Histotype**	**Number of diagnoses**
Benign mixed tumour	4237
Adenoma, complex	3303
Carcinoma, complex	1714
Carcinoma, simple	1531
Carcinoma, ductal	1139
Adenoma, simple	928
Adenoma, ductal, papillary	878
Adenoma, ductal	692
Carcinoma, simple, tubular	463
Carcinoma, mixed	374
Carcinoma, ductal, papillary	368
Carcinoma, simple, solid	304
Carcinoma, simple, tubulopapillary	269
Carcinoma arising in a complex adenoma/benign mixed tumour	158
Carcinoma, simple, anaplastic	135
Adenoma, simple, tubular	133
Carcinoma, simple, comedo	108
Carcinosarcoma	70
Carcinoma, simple, papillary	64
Adenoma, simple, tubulopapillary	54
Carcinoma and malignant myoepithelioma	44
Carcinoma, simple, tubular to solid	43
Carcinoma, simple, micropapillary	42
Carcinoma, adenosquamous	33
Carcinoma, lipid-rich	32
Malignant myoepithelioma	21
Adenoma, simple, papillary	20
Myoepithelioma	17
Carcinoma, simple, cribriform	16
Carcinoma, inflammatory	15
Carcinoma, spindle cell	13
Carcinoma, simple, tubulopapillary to solid^a^	8
Carcinoma, simple, solid to comedo^a^	6
Carcinoma, simple, solid to tubular^a^	5
Carcinoma, simple, tubular to solid to comedo^a^	3
Carcinoma, simple, tubulopapillary to comedo^a^	3
Carcinoma, complex to micropapillary^a^	2
Carcinoma, mucinous	2
Carcinoma, simple, solid to anaplastic^a^	2
Carcinoma, simple, solid to micropapillary^a^	2
Carcinoma, simple, tubular to anaplastic^a^	2
Carcinoma, simple, tubulopapillary to anaplastic^a^	2
Adenoma, simple to complex to ductal, papillary^a^	1
Adenoma, simple, tubular to complex^a^	1
Carcinoma, complex to adenosquamous^a^	1
Carcinoma, complex to tubulopapillary^a^	1
Carcinoma, simple, cribriform to solid to comedo^a^	1
Carcinoma, simple, papillary to anaplastic^a^	1
Carcinoma, simple, solid to adenosquamous^a^	1
Carcinoma, simple, solid to comedo to anaplastic^a^	1
Carcinoma, simple, solid to complex^a^	1
Carcinoma, simple, solid to cribriform^a^	1
Carcinoma, simple, solid to cribriform to comedo^a^	1
Carcinoma, simple, solid to spindle cell^a^	1
Carcinoma, simple, solid to tubular to comedo^a^	1
Carcinoma, simple, solid to tubulopapillary to comedo^a^	1
Carcinoma, simple, solid, cystic	1
Carcinoma, simple, tubular to comedo^a^	1
Carcinoma, simple, tubular to cribriform^a^	1
Carcinoma, simple, tubular to mixed^a^	1
Carcinoma, simple, tubular to mucinous^a^	1
Carcinoma, simple, tubular to spindle cell^a^	1

^a^ Intermediate forms

**Table 2:** Percentage of tumour histotypes diagnosed in cases with single lesions

**Histotype**	**Number of cases**	**Percent of cases**
Benign mixed tumour	2126	23.55%
Adenoma, complex	1340	14.84%
Carcinoma, simple	1028	11.39%
Carcinoma, complex	970	10.74%
Carcinoma, ductal	722	8.00%
Adenoma, ductal, papillary	391	4.33%
Carcinoma, simple, tubular	313	3.47%
Adenoma, ductal	309	3.42%
Adenoma, simple	293	3.25%
Carcinoma, ductal, papillary	234	2.59%
Carcinoma, mixed	233	2.58%
Carcinoma, simple, solid	220	2.44%
Carcinoma, simple, tubulopapillary	180	1.99%
Carcinoma, simple, anaplastic	102	1.13%
Carcinoma arising in a complex adenoma/benign mixed tumour	89	0.99%
Carcinoma, simple, comedo	84	0.93%
Carcinosarcoma	53	0.59%
Adenoma, simple, tubular	47	0.52%
Carcinoma, simple, papillary	46	0.51%
Carcinoma and malignant myoepithelioma	33	0.37%
Carcinoma, lipid-rich	30	0.33%
Carcinoma, simple, micropapillary	27	0.30%
Adenoma, simple, tubulopapillary	24	0.27%
Carcinoma, adenosquamous	21	0.23%
Carcinoma, inflammatory	12	0.13%
Carcinoma, spindle cell	9	0.10%
Malignant myoepithelioma	8	0.09%
Adenoma, simple, papillary	7	0.08%
Carcinoma, simple, cribriform	7	0.08%
Myoepithelioma	5	0.06%
Carcinoma, mucinous	1	0.01%
Intermediate forms	65	0.72%

### Risk Factors Associated with Clinical Features of Mammary Tumours

In univariable analysis, there was a significant association of *breed* (*p* < 0.0001), *age* (*p* < 0.0001) and *neuter status* (*p* < 0.0001) with odds of mammary tumour classification as malignant (Table S7). All three were therefore taken forward into a multivariable model, where again all three were significantly associated with risk of malignant disease.

**Fig. 3:**
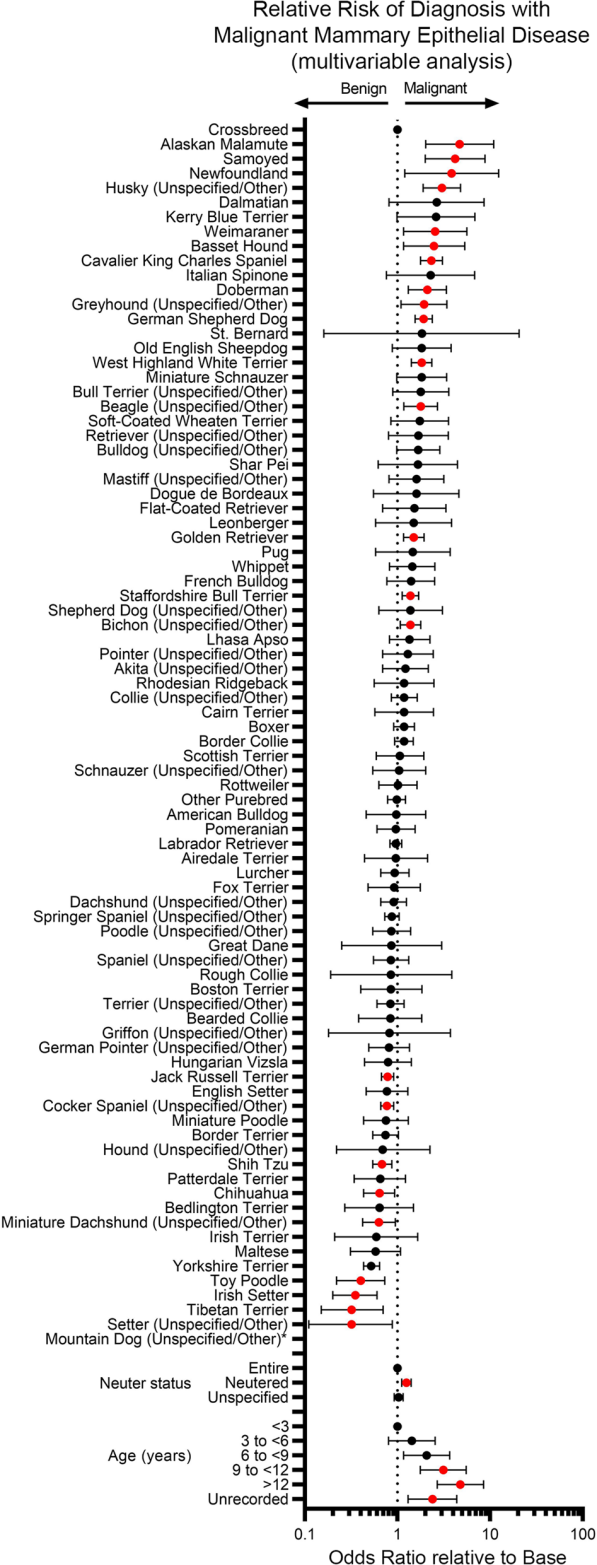
Correlation of features of study population with risk of malignancy of neoplastic epithelial mammary lesions. Forest plot showing results of multivariable analysis of *breed*, *neuter status* and *age* relationship of study population to odds of a diagnosis of a malignant mammary epithelial neoplasm. Odds ratios and 95% confidence intervals are shown. Variables showing a significant difference from base reference value (Crossbreed for *breed*, Entire for *neuter status*, < 3 years for *age*) indicated in red. See also Table S7. *Odds Ratio with 95% confidence intervals cannot be provided for Mountain Dog (Unspecified/Other) as all five cases in this breed were malignant. Confidence intervals are very wide for breeds such as the St Bernard as they are very rare breeds in the data set

For *age* (*p* < 0.0001 overall), dogs presenting at older ages had significantly higher odds of a diagnosis of malignant disease relative to younger dogs (6 to < 9 years: OR 2.06, 95%CI 1.16—3.65; 9 to < 12 years: OR 3.12, 95% CI 1.76—5.52; > 12 years: OR 4.77, 95%CI 2.68—8.48). For *neuter status* (*p* < 0.0001 overall), neutered animals were significantly more likely to have a diagnosis of malignant disease relative to entire bitches (OR 1.25, 95%CI 1.11—1.4) (Table S7 and Fig. 3).

Several breeds had significantly higher or lower odds of a diagnosis of malignant mammary epithelial disease relative to Crossbreeds in the multivariable analysis. In particular, higher odds (ORs from 4.69 to 1.38, *p* < 0.05) were seen in the Alaskan Malamute, Samoyed, Newfoundland, Husky, Weimaraner, Basset Hound, Cavalier King Charles Spaniel, Doberman, Greyhound, German Shepherd Dog, West Highland White Terrier, Beagle, Golden Retriever, Bichon and the Staffordshire Bull Terrier. Lower odds (ORs from 0.78 to 0.32, *p* < 0.05) were seen in the Jack Russell Terrier, Cocker Spaniel, Shih Tzu, Chihuahua, Miniature Dachshund, Yorkshire Terrier, Toy Poodle, Irish Setter, Setter and the Tibetan Terrier (Table S7 and Fig. 3).

There was a significant association in univariable analysis of *breed* (*p* < 0.0001), *age* (*p* < 0.0001) and *neuter status* (*p* < 0.0001) with a case being diagnosed with multiple versus single mammary epithelial neoplastic lesions (Table S7). All three were therefore taken forward into a multivariable model. All three were significantly associated with altered odds of diagnosis of multiple lesions in the multivariable model.

**Fig. 4:**
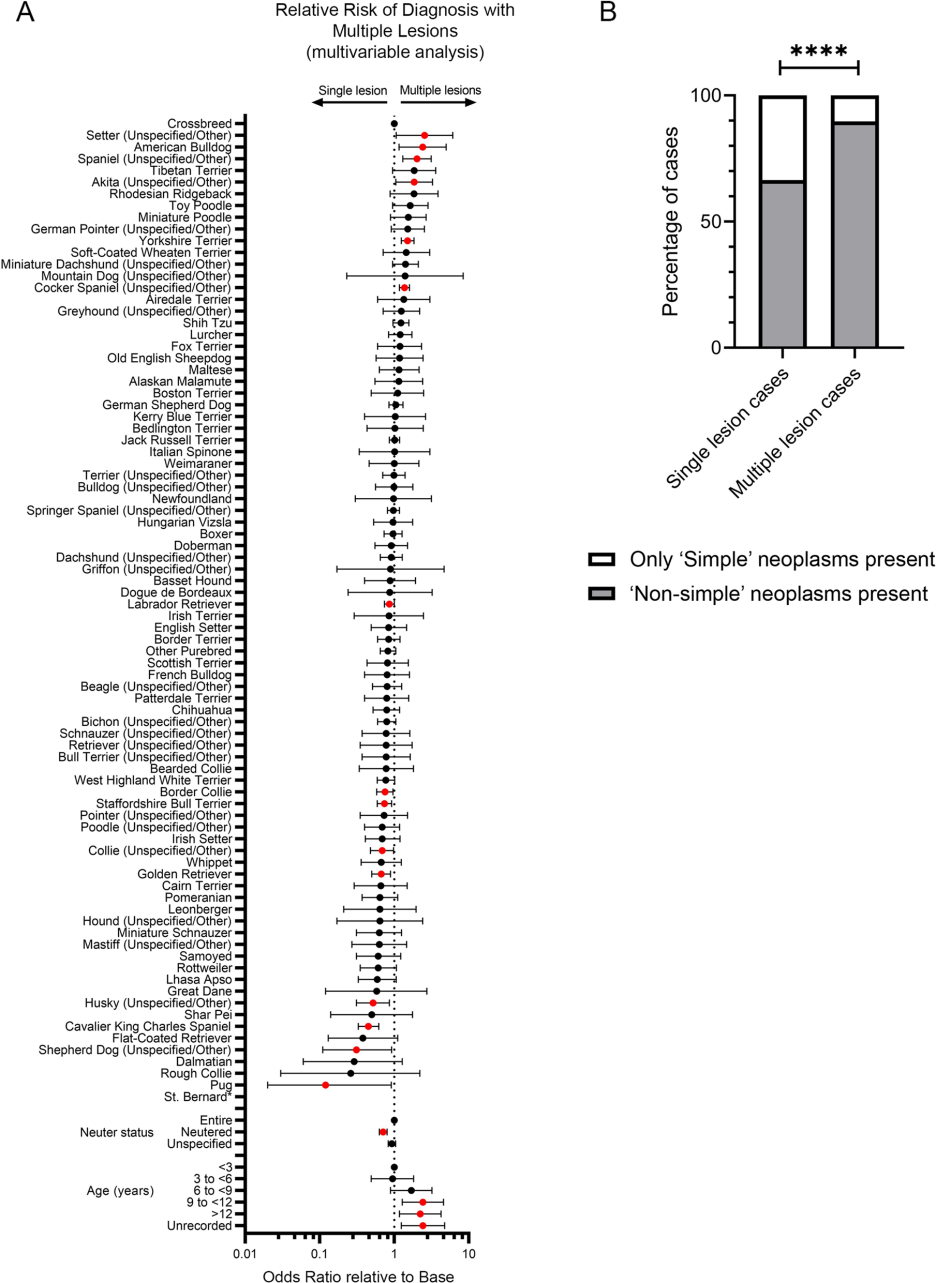
Correlation of features of study population with risk of presenting with multiple neoplastic epithelial mammary lesions. **A** Forest plot showing results of multivariable analysis of *breed*, *neuter status* and *age* relationship of study population to odds of being diagnosed with multiple mammary epithelial neoplasms. Odds ratios and 95% confidence intervals are shown. Variables showing a significant difference from base reference value (Crossbreed for *breed*, Entire for *neuter status*, < 3 years for *age*) indicated in red. See also Table S7. *Odds Ratio with 95% confidence intervals cannot be provided for the St Bernard as all three cases in this breed were single cases. **B** Proportion of cases presenting with single and multiple lesions diagnosed with one or more Non-Simple neoplasms, as opposed to only Simple neoplasms. ****p < 0.0001 (Fisher’s exact test)

For *age* (*p* < 0.0001 overall), dogs presenting at older ages had a significantly higher odds of a being diagnosed with multiple lesions compared to younger dogs (9 to < 12 years: OR 2.42, 95%CI 1.28—4.59; > 12 years: OR 2.23, 95%CI 1.17—4.25). For neuter status (*p* < 0.0001 overall), neutered animals had significantly lower odds of being diagnosed with multiple lesions than entire bitches (OR 0.71, 95%CI 0.63—0.8) (Table S7 and Fig. 4A).

A number of breeds had significantly higher or lower odds of being diagnosed with multiple lesions relative to Crossbreeds in the multivariable analysis. Higher odds (ORs from 2.55 to 1.37, *p* < 0.05) were seen in the Setter, American Bulldog, Spaniel, Akita, Yorkshire Terrier and Cocker Spaniel. Lower odds (ORs from 0.75 to 0.12, *p* < 0.05) were seen in the Border Collie, Staffordshire Bull Terrier, Collie, Golden Retriever, Husky, Cavalier King Charles Spaniel, Shepherd Dog and Pug (Table S7 and Fig. 4A).

The changes in ORs of developing malignant / multiple neoplasms relative to baseline are summarised for the *breed*, *age* and *neuter status* risk factors in Table S9.

### Non-simple Histotypes are Significantly more Likely to be Found in Tumours from Cases Presenting with Multiple Lesions

Mammary epithelial neoplastic lesions are classified into simple, non-simple, ductal and special histotypes [35]. Non-simple neoplasms contain proliferating neoplastic populations of more than one lineage, including proliferating luminal epithelial and myoepithelial cells and potentially mesenchymal elements. We speculated whether cases which present with multiple neoplasms are more likely to have a higher proportion of non-simple neoplasms, composed of multiple cellular lineages, because of a generalised increased risk of carcinogenesis across all cells in the entire mammary chain. Therefore, we assessed whether such non-simple lesions were more frequent in animals diagnosed with multiple lesions. Considering only cases in which either simple or non-simple lesions were diagnosed, either alone or in combination with other types (excluding those cases in which only ductal lesions, special types or intermediate forms were diagnosed) (11,459 cases), non-simple lesions were identified significantly more frequently (89.74% of cases) when multiple lesions were diagnosed compared to cases with only single lesions (66.48% of which were non-simple) (Fisher’s exact test of categorical variables, *p* < 0.0001) (Fig. 4B).

## Discussion

Here, we have analysed histological diagnoses from an archive of 129,258 records of submissions of samples from bitches, including 13,401 mammary epithelial neoplasms, sent for histological assessment to a single histopathology laboratory between 2008 and 2021. In multivariable analysis, we found a significant association between *breed*, *age* and *neuter status* and the odds that a sample submitted for histological diagnosis would be diagnosed as a mammary epithelial tumour, as opposed to any other diagnosis. We also found a significant association in multivariable analysis between *breed*, *age* and *neuter status* and odds of diagnosis of a malignant versus benign mammary epithelial neoplasm and also the odds of being diagnosed with multiple versus single mammary epithelial neoplasms. Our study was not designed to determine how *breed*, *age* and *neuter status* affect odds of mammary tumour diagnosis relative to the general canine population as this requires comparison to the entire population of canines in a particular region as a denominator. We (D. Varney, D. O'Neill, M. O'Neill, D. Church, A. Stell, S. Beck, M. Smalley and D. Brodbelt: The epidemiology of mammary tumours in bitches under veterinary care in the UK in 2016, submitted) and others [3, 7, 9, 24–27] have, however, addressed this question elsewhere. Our study was primarily designed to address how these variables might influence clinical features of tumours once histologically diagnosed; however, we also took advantage of our dataset to understand how they affect the likelihood that any sample undergoing histological assessment was diagnosed as a mammary epithelial neoplasm rather than any other histological diagnosis.

An increased likelihood of a sample submitted being histologically diagnosed as a mammary epithelial tumour in older animals reflects mammary neoplasia (and in particular adenoma/ adenocarcinoma) as a disease of older dogs [29, 43]. Similarly, an increased likelihood in entire animals would be consistent with studies showing that neutered animals are protected against mammary neoplasia [11–13, 44–47] (Varney D, O'Neill D, O'Neill M, Church D, Stell A, Beck S, Smalley M, Brodbelt D: The epidemiology of mammary tumours in bitches under veterinary care in the UK in 2016, submitted). However, caution should be exercised in the interpretation of our results, as our findings specifically relate to samples submitted for histological examination and sample submission bias is likely to be a factor. This is discussed further below.

Using the large dataset, of 13,401 cases submitted with one or more confirmed epithelial-origin mammary neoplastic lesions (sometimes in addition to other pathologies) available to us, we next asked whether the *age*, *neuter status* and *breed* variables were also associated with the clinical behaviour of the mammary neoplasms in these cases. We examined the odds of developing a malignant (as opposed to benign) lesion and the odds of being diagnosed with multiple (as opposed to single) lesions. In all cases, our analysis was based on odds compared to a baseline group (age < 3 years; ‘entire’ neuter status and Crossbreed animals) (summarised in Table S9).

A number of studies have assessed the relationship between age at diagnosis and the risk of being diagnosed with benign or malignant mammary lesions [7, 20, 26, 27, 48–50] and of being diagnosed with single or multiple mammary lesions [26] (although the proportion of dogs with single or multiple lesions varies widely between reports, potentially a result of differing proportions of breeds within the study populations) [49, 51]. We find in multivariable analysis that older dogs are more likely to be diagnosed with malignant disease and with multiple lesions. This is consistent with previous reports that dogs with malignant tumours are more likely to be older than dogs with benign tumours [26, 48, 49], that age is an independent prognostic factor correlating with poor survival [20] and that older animals tend to have higher grade ER negative tumours [27]. A number of mechanisms may drive this increased risk of malignancy in older dogs. For example, it may simply be a stochastic process, with more mutations required to generate a malignant tumour than a benign one. An example of this may be carcinoma arising in complex adenoma/benign mixed tumour. Cellular aging may also be a factor as a result of telomere shortening, which would lead to increased risk of genomic abnormalities [52]. Finally, older individuals tend to have a dysfunctional immune system [53], with potential for reduced immunosurveillance permitting tumour growth and metastasis [54].

Neuter status is also well established as a risk factor for development of mammary epithelial neoplasia. Multiple studies, including our own, have demonstrated that neutered bitches have a reduced risk of neoplastic mammary disease compared to entire animals [10, 11, 44–47] (summarised in [13]). Here, we have now shown in multivariable analysis that neutered animals are also at reduced odds of developing multiple lesions; however, if they do develop neoplastic mammary disease, they are at increased odds that this will be malignant. It is notable that ER negative canine mammary tumours (CMT) are more likely to be malignant than ER positive CMT [16–18]. Furthermore, a study which examined the link between neutering, serum estrogen levels and CMT hormone receptor status demonstrated that ER negative tumours in entire animals with high serum hormone levels had a longer time to metastasis than such tumours in neutered animals [15]. A protective effect of estrogen via non-receptor mechanisms was suggested.

Some studies have found no links between breed and predisposition to develop malignant as opposed to benign lesions [50] and no difference in malignancy between cases presenting with single or multiple masses [55]. However, others have suggested particular breeds are more likely to develop malignant mammary tumours (Samoyed, Dobermann, Schnauzer and Yorkshire Terrier) [56] (although notably when cancer of all sites was considered Aupperle-Lellbach and colleagues found Yorkshire Terriers among the breeds more likely to develop benign tumours [24]). There is little, if any, information on the link between breed and risk of developing single as opposed to multiple lesions. We find a significant association between breed and both of these aspects of tumour biology. The patterns of odds of developing malignant disease, or multiple lesions, across individual breeds showed that no breed was found to be at increased odds of both outcomes. They were either at increased odds of malignant disease and decreased odds of multiple lesions (e.g. King Charles Cavalier Spaniel, Golden Retriever, Husky, Staffordshire Bull Terrier) or vice versa (e.g. Cocker Spaniel, Setter, Yorkshire Terrier) or they were at significantly altered odds of one outcome with no change in the other outcome.

The simultaneous appearance of multiple mammary epithelial neoplasms in cases presenting with multiple lesions suggests a ‘field cancerisation’ model. Field cancerisation was first proposed by Slaughter and colleagues in 1953, to describe the development, in oral squamous cell carcinoma, of regions of the oral epithelium which are clinically apparently normal but in which multiple independent primary squamous cell carcinomas continually arise [57]. It is now considered that field cancerisation is the product of an underlying preneoplastic stem cell which has acquired mutations giving it a competitive advantage overing neighbouring cells, allowing clonal progeny of the original preneoplastic cell to spread. This then creates a large target population requiring fewer mutational events for full transformation [57–61]. We suggest that the presence of multiple independent tumours, a high proportion of which are non-simple tumours composed of multiple cell lineages, supports a model that in some dogs the entire mammary epithelium is a field of preneoplastic (stem) cells. Furthermore, we suggest that there can be genetic factors predisposing to the development of a preneoplastic mammary field. A number of candidate genes have been identified which affect cell competition and have known roles in cancer, for example p53 [60], so these would be an excellent starting point for future studies.

Interestingly, Gunnes and colleagues [49] reported that in bitches presenting with multiple tumours, the chance that two tumours would have the same diagnosis and level of malignancy was greater than would be expected by chance alone, leading them to conclude the existence of a hormone-driven field cancerisation effect. They also suggest that there might be breed variations in predisposition to malignant (as opposed to benign) tumours but their study was not sufficiently powered to identify specific at-risk breeds.

Links between canine mammary tumour histological subtype and prognosis are well established [19–22] and the prevalence of different histological subtypes within canine mammary neoplasia has also been assessed in a number of studies, for example [7, 49, 50]. Results differ widely from study to study, likely as a result of differing study populations and differing histological interpretations. Salas and colleagues report similar frequencies of benign and malignant tumours in their study, with more epithelial-type than mixed neoplasms [7]; Gunnes and colleagues reported 61% of examined tumours were benign, 39% were malignant, with complex adenoma and complex carcinoma the most frequent diagnosis in each category [49]; Ariyarathna and colleagues report 56% of examined tumours as malignant (simple carcinomas being most common) and 44% benign (benign mixed tumours being most common). We find that in cases with single lesions being diagnosed, the most common lesions were the benign mixed tumour (23.6%), adenoma, complex (14.8%), carcinoma, simple (subtype not otherwise specified (11.4%), carcinoma, complex (10.7%) and carcinoma, ductal (8.0%).

While a diagnosis of mixed and complex (non-simple) mammary neoplasms is common in the dog, no studies have yet reported a link between a diagnosis of non-simple tumours and development of multiple lesions. The link between complex/mixed lesions and presentation with multiple lesions (with a potential field cancerisation effect) is of particular interest as the origin of complex/mixed lesions remains unclear. Complex tumours contain distinct proliferating luminal epithelial and myoepithelial populations [35] Mixed tumours contain, in addition, mesenchymal elements [35]. It is possible that complex/mixed tumours are polyclonal in origin, with separate transformed luminal and myoepithelial cells (in the case of a complex tumour) or luminal, myoepithelial and mesenchymal stem cells (in the case of mixed tumours) all contributing to a neoplasm presumably in response to a highly localised tumour promoting factor, such as an inflammatory signal. Alternatively, these tumours might arise monoclonally from a mammary epithelial stem cell capable of undergoing both luminal and myoepithelial differentiation as well as metaplastic potential. Previous studies have addressed this question by analysis of mitochondrial DNA mutations in the epithelial and mesenchymal elements [62] or by analysis of immunohistochemical staining patterns and DNA ploidy of the different components of the tumour [63]. These analyses have suggested some tumours may be polyclonal while others may be monoclonal, but many of the analyses have not been informative. A definitive answer on the aetiology of these tumours awaits further study.

Our analysis supports the hypothesis that neuter status, age and intrinsic biological and genetic factors all influence the heterogeneity of clinical presentation of canine mammary neoplasia. The term ‘heterogeneity’ includes both ‘intra-tumour heterogeneity’, the genetic, epigenetic, phenotypic and/or behavioural differences in cells within a tumour (including both neoplastic cells and non-neoplastic cells such as tumour-associated fibroblasts and macrophages), and inter-tumour heterogeneity, the classification of tumours as different histotypes which may have different clinical behaviours (e.g. benign or malignant disease) and approaches to therapy [64]. Tumour heterogeneity may be considered to arise from two processes. In the early phase of tumour development, it arises from the interaction between cell of origin of the tumour and the initiating genetic lesions occurring in that tumour [65–67]. Then, as the tumour progresses, the random generation of progressively more mutated clones, combined with selective pressures on these variant clones, lead to tumour heterogeneity through a Darwinian evolutionary process [68]. We would argue that the former process is the main driver of inter-tumour heterogeneity while the latter is the main driver of intra-tumour heterogeneity. Of course, these are not clear-cut divisions, considering that a change in the proportion of different cell types within a tumour (for example, cells that express hormone receptors in a mammary neoplasm) could lead to a breast cancer being reclassified as progressing from ER positive to ER negative disease i.e. a change in intra-tumour heterogeneity leads to a change in inter-tumour heterogeneity.

Modelling the interaction between the cell of origin and genetic lesion as a determinant of mammary tumour heterogeneity in genetically modified mice has confirmed the principles that tumour histotype is driven by the interactions between cell of tumour origin, initiating genetic lesion and in some cases developmental history of the gland [65–67, 69]. However, this is an artificial system in which candidate genes are bred into mice in conditional knockout/overexpression scenarios and in which a limited number of tumour histotypes develop with (for the most part) little clinical relevance either to human or veterinary medicine. In contrast, canine mammary tumours combined with the power of dog genetics offer a system in which, rather than choosing the genes of interest and working forward to understand what, if any, effect they have on tumour phenotype, one can work backwards from tumour phenotype to elucidate the underlying genetics. The first stage of this is to use an epidemiological approach to establish associations between tumour biology and breed and develop hypotheses. These can then be tested in case–control genomic studies within and between breeds [70] to identify loci associated with the particular aspect of biology (e.g. presentation with multiple tumours or a diagnosis of malignant disease) which can be taken forward into mechanistic studies. The results we present here represent the first stage of this process.

Our study has limitations and caveats, the principle one being selection bias (i.e. the dogs in the current study may not be representative of the general caseload of canine mammary tumours as a whole) [71]. There are many factors which affect clinical decision making when deciding whether to submit lesions for histological analysis and these could all bias the population of dogs that appear in the current study ( a ‘biopsy-only’ dataset). For example, dogs with very severe disease or poorly resectable lesions may undergo euthanasia or palliative care rather than surgical resection and histological analysis. In contrast, an entire bitch which presents at a primary care practice with a mammary mass may be more likely to have that mass sent for histology than a neutered bitch, simply because of the previously postulated links between neuter status and mammary tumour risk. Furthermore, financial limitations regarding the cost of surgery and biopsy submission may mean that dogs owned by owners of lower socio-economic status or uninsured dogs may be less likely to appear in the current dataset. Previous studies have shown that both breed and neutering status in dogs are associated with owner socioeconomic status, therefore if owner demographics are biased in the dataset, the distribution of breeds or neuter status may be altered [4]. The socio-economic circumstances or age of owners may also have resulted in an underrepresentation of benign lesions, for example, if individuals with lower economic means or who face challenges accessing veterinary care were less inclined to take a dog with a mammary mass for veterinary attention unless it shows obvious signs of malignancy, such as extremely rapid growth or obvious morbidity. Furthermore, in certain breeds a single benign lesion may be more difficult for an owner to detect owing to body shape or conformation, whereas multiple lesions may be more likely to lead to an owner seeking attention. We found an inverse correlation between average breed body weight and likelihood of a histopathological sample being diagnosed as a mammary epithelial tumour. However, while a predisposition for mammary tumour development in small dogs has been reported [7, 29] population-based case–control studies have identified both small and large breeds as being at higher odds of developing mammary epithelial neoplasia [3, 7, 9, 24–26]. It may be that smaller dogs are more likely to be picked up and have mammary masses discovered by owners. Another alternative is that small dogs live longer than large dogs, and mammary neoplasia is a disease of older animals. Overall, selection bias could have affected the observed associations between *breed*, *age* and *neuter status* and mammary tumour number or histotype.

The study may also have been affected by missing data because in veterinary clinical practice, there is not a standardised approach to lumpectomy vs mammectomy vs full mammary strip removal. While full mammary strips were frequently submitted (and indeed such submissions were encouraged), we cannot exclude that some practices did not submit all tissue or all lesions present in a dog for analysis, leading to reporting of cases with more than one lesion as single lesion cases. However, it is more likely that in cases which present with only one, or a small number of, palpable lesions, all will be sent for analysis, whereas in cases presenting with many such lesions only a representative sample might be examined. Such an approach would still result in the case being correctly categorised as having multiple lesions. It is more likely that cases could be incorrectly categorised as having single lesions, if only a single palpable lesion is detected and sampled, but small, clinically undetectable, lesions are already present.

Once tissue has been submitted, given the extensive experience of VPG Histopathology, it is highly unlikely that cases would be incorrectly categorised as ‘benign’ rather than ‘malignant’, even during histological analysis of large pieces of tissue (such as whole mammary strips) owing to the meticulous nature of the analysis. However, we cannot definitively exclude that occult malignant cell clusters may be present in an otherwise benign tissue and, if small, these cases could theoretically be miscategorised as benign-only.

We did not carry out Bonferroni corrections for multiple comparisons, which may have caused some Type-1 errors. However, this is usually considered overly stringent where there may be correlation between variables [72]. Furthermore, although unlikely, we cannot exclude the possibility that there may be multiple samples submitted for histological examination from the same animal on different occasions. To definitively exclude this would require access to information that would identify owners and therefore such data was withheld from the current study.

We also did not account for the expected breed lifespan when considering risk factors for cancer. However, *breed* and *neuter status* may affect the years-at-risk of dogs, and thus certain breeds with longer lifespans, or neutered dogs (shown to live longer on average) may appear to be more at risk simply because they experience more years-at risk of disease. The inclusion of this complex variable was beyond the scope of this study because a reliable published lifespan could not be sourced for all of the included breeds, however future analyses considering years-at-risk would be valuable for validation of the conclusions presented here.

Importantly, our study represents only a snapshot in the clinical pathway of each animal diagnosed. The diagnostic records available to us are not linked to clinical outcome, so we do not know whether a diagnosis of single or multiple lesions affects prognosis although it is clear that malignant disease has a worse prognosis than benign disease (the prognostic significance of histological subtypes is also established) [19, 20]. Both benign and malignant disease was seen in cases diagnosed with both single and multiple lesions, and previous findings have also shown no relationship between the presence of single and multiple masses and a malignant diagnosis [55]. It is unclear whether having multiple lesions is an independent prognostic factor. To test this would require a prospective study in order to ensure that potential confounding factors are controlled for (e.g. the lack of a uniform approach to treating mammary masses). Testing this would be an important follow-up to the current study.

Our study also only represents a snapshot of the underlying biology of the disease. We cannot exclude, for example, that a bitch diagnosed with a single malignant lesion might have, in the future, gone on to develop multiple lesions, or that an animal with multiple lesions that has a full mammary strip, and all those lesions are diagnosed as benign after surgery, might have developed a malignant tumour at a later date if no interventions were performed. To draw definitive conclusions that a case with a single lesion diagnosis would not have later been diagnosed with multiple lesions, or that a case with one or more benign lesions would not have later been diagnosed with one or more malignant lesions would require surgeons to perform only individual lumpectomies on dogs presenting with mammary masses, no matter how many masses they present with and no matter how many times they return to clinic. This is not consistent with the best welfare of the animals involved. However, as our study sample overall captures a very large population over a period of years (canine lifespans), generalisations seem reasonable, as the specific sampling time in the course of the disease becomes less important for each individual.

Therefore, although the caveats above must be kept in mind, we have found significant associations between the *breed*, *age* and *neuter status* of a bitch, and whether a presentation for mammary neoplasia is likely to be for single lesions or multiple lesions, and whether those lesions are likely to be benign or malignant. Furthermore, non-simple lesions are enriched in cases presented with multiple neoplasms. We therefore suggest that underlying genetic factors can affect tumour heterogeneity, by influencing clinical behaviour (the development of benign or malignant disease), tumour number and cellular composition. Environmental influences such as aging are also likely to play a role. The mammary epithelium of breeds at higher risk of presenting with multiple neoplastic mammary lesions may be a pre-neoplastic field genetically primed for tumour development. Case–control genomic studies, and mechanistic evaluation, have the potential to identify, in an unbiased manner, genes driving mammary tumour behaviours and thus such studies could substantially advance our understanding of the drivers of mammary tumour formation and tumour heterogeneity and, ultimately, identify new targets for therapy.

## Supplementary Information

**Additional file 1: Supplementary Table S1.** Signalment of the complete data set including VPG Histopathology Case Number, Case Breed (the original entry from the VPG database), the consolidated and simplified breed terms and the final breed term used in this analysis (in which breeds with <130 cases were classified as ‘Other Purebred’). The age in years and months, the sex and neuter status, the classification of the case as a diagnosis of a mammary epithelial neoplasm (no/yes/triaged out) and the histological diagnosis are provided.

**Additional file 2: Supplementary Table S2.** Full analysis of cases diagnosed with mammary epithelial neoplasia, including breed, age, sex and neuter status and full analysis and classification of mammary neoplasia. Whether or not the case had a malignant neoplasm (yes/no) and multiple neoplasms (yes/no) are indicated as well as whether or not ductal, simple, non-simple, special and intermediate neoplastic lesions were present (yes/no).

**Additional file 3: Supplementary Table S3.** Categorisation of mammary epithelial neoplasia histological subtypes based on the classification in Surgical Pathology of Domestic Animals Volume 2: Mammary Tumors [35].

**Additional file 4: Supplementary Table S4.** Information used for regression analysis of relative odds of diagnosis with a mammary epithelial neoplasm (extracted from Table S1).

**Additional file 5: Supplementary Table S5.** Information used for regression analysis of relative odds of diagnosis with a malignant mammary epithelial neoplasm or of being diagnosed with multiple lesions (extracted from Table S2).

**Additional file 6: Supplementary Table S6.** Descriptive statistics of each case used for regression analysis.

**Additional file 7: Supplementary Table S7.** Outcomes of univariable and multivariable regression analyses of differential odds of being diagnosed with a mammary epithelial neoplasm (as opposed to any other diagnosis), of being diagnosed with malignant (as opposed to benign) disease and of presenting with multiple (as opposed to single) lesions.

**Additional file 8: Supplementary Table S8.** Mean weight for females >18 months old for breeds where information is available breed and the mean body weight for a female of that breed (taken from VetCompass estimates of average mass) [30, 42]. The odds of being diagnosed with a mammary epithelial neoplasm (as opposed to any other diagnosis) are also provided (from Table S7).

**Additional file 9: Supplementary Table S9.** Summary table indicating relationship between age, neuter status and breeds and associations with increased or decreased odds of developing malignant disease or being diagnosed with multiple lesions, relative to the baseline for that analysis. Only categories within each risk factor (e.g. individual breeds) which showed a significant increase or decrease in odds relative to baseline in at least one of the two categories are shown.

**Additional file 10: Supplementary Data File.** Python script as a Jupyter Notebook (.ipynb) file for automated extraction of pathology features. The script can be uploaded to a Kaggle notebook (www.kaggle.com) and used to extract features from data uploaded as an Excel spreadsheet. Data files uploaded for analysis should be in the form of six columns with the following headers: CaseNumber; CaseBreed; CaseAnimalAgeYears; CaseAnimalAgeMonths; CaseSex; CaseHistologicalDiagnosis.
